# Assessment of knowledge on neonatal resuscitation amongst health care providers in Kenya

**Published:** 2012-04-24

**Authors:** Florence Murila, Moses Madadi Obimbo, Rachel Musoke

**Affiliations:** 1 University of Nairobi, Kenya

**Keywords:** Neonatal resuscitation, knowledge, training, Kenya, Africa

## Abstract

**Introduction:**

Competence in neonatal resuscitation, which represents the most urgent pediatric clinical situation, is critical in delivery rooms to ensure safety and health of newly born infants. The challenges experienced by health care providers during this procedure are unique due to different causes of cardio respiratory arrest. This study aimed at assessing the knowledge of health providers on neonatal resuscitation.

**Methods:**

Data were gathered among 192 health providers drawn from all counties of Kenya. The clinicians were asked to complete questionnaires which were in two parts as; demographic information and assessment of their knowledge by different scenarios which were formatted in the multiple choice questions. Data were analyzed using SPSS version 15.0 for windows. The results are presented using tables.

**Results:**

All the participants were aged 23 years and above with at least a certificate training. Most medical providers had heard of neonatal resuscitation (85.4%) with only 23 receiving formal training. The average duration of neonatal training was 3 hours with 50% having missed out on practical exposure. When asked on steps of resuscitation, only 68 (35.4%) of the participants scored above 85%. More than 70% of them considered their knowledge about neonatal resuscitation inadequate and blamed it on inadequate medical training programs.

**Conclusion:**

Health providers, as the key personnel in the management of neonatal resuscitation, in this survey seem to have inadequate training and knowledge on this subject. Increasing the duration and quality of formal training should be considered during the pre-service medical education to ensure acceptable neonatal outcome.

## Introduction

Effective newborn resuscitation is essential in reducing the sequel of birth asphyxia estimated to have a mortality of 2 million a year with 99% of deaths in developing countries [1, 2]. For resuscitation to be successful, it requires meticulous understanding by the health-care personnel working in the labour, maternity and newborn units to have adequate skills for prompt neonatal resuscitation [3, 4]. Adequate knowledge and awareness about neonatal resuscitation plays a major role in early diagnosis, appropriate management and, accordingly, reduction of adverse consequences [5]. Some countries have well established neonatal resuscitation programs aimed at equipping their health personnel with skills for neonatal resuscitation [6–8]. In Kenya, adoption and implementation of such programs remains a challenge. The aim of this study was the determine knowledge level about neonatal resuscitation amongst health care personnel working in Kenyan hospitals.

## Methods

The study consisted of 192 health care personnel drawn from all the counties of Republic of Kenya. Kenya has 47 counties that represent administrative units. Each county has either a level IV or V hospital with an active neonatal unit. The average workforce in each neonatal unit of county hospital is made of eight to ten medics (nurses and clinicians). Four medics working in these neonatal units were randomly selected from each county hospital. Two referral hospitals in Uasin Gishu and Nairobi counties were each assigned six medics because of the large volumes of patients that they attend to and the larger number of clinicians. The administrative authority to carry out the study was given by the Ministry of Medical Services of Kenya. Data on their age, sex, experience in medical service, academic qualifications, work station and previous training in neonatal resuscitation was collected and filled in a preformatted questionnaire. A written test with 10 questions derived from the standard test contained in the American Heart Association/ American Academy of Paediatrics (AHA/AAP) *Textbook of Neonatal Resuscitation* [3, 9] was given to them. The questions were divided into the five steps according to the stages of neonatal resuscitation: (i) initial steps in resuscitation; (ii) ventilation; (iii) chest compression; (iv) endotracheal intubation; and (v) administration of medications and fluids. The participants took the examination under standardized conditions, strictly supervised, and were required to complete the test in 20 minutes. The questions included multiple choice and true/false questions. The grading of the test was carried out according to standard instructions contained in the *Textbook of Neonatal Resuscitation*. Those who scored >85 were scored as successful.

Data were managed and analyzed by Statistical program for Social Sciences (SPSS) 15.0 Chicago Ill software. All numeric values are expressed exact number (n) or percentage (%). Categorical variables were compared using X^*2*^ test. P

## Results

Participants included 109 females and 83 males all aged above 23 years (with average age of 35 years) and at least a certificate training (Table 1). The average duration of work experience was 9 years. Majority of the participants (71%) did not have an experience with neonatal resuscitation.

**Table 1:** Participants in the survey

Professional	Highest qualification	Number (%)
Medical officer	Degree	28 (14.6)
Clinical officer	Diploma	34 (17.7)
Nursing officer	Certificate	112 (58.3)
	Degree	18 (9.4)

One hundred and sixty three health care personnel (85%) had received some information on neonatal resuscitation. Out of this number only 23 (12%) had formal training. Of those who received formal training, the total course duration averaged 3 hours with almost 50% missing out on practical exposure. When asked on initial steps in resuscitation, ventilation, chest compression, endotracheal intubation and administration of medications and fluids only 68 participants (35.4%) obtained a score of 85% and above, the rest getting them wrong (Table 2). When the average scores of the nurses was compared with that of the clinicians using X^2^test, the differences were found to be statistically significant (p=0.046) in favour of clinicians. More than 70% of the participants considered their knowledge about neonatal resuscitation less than average. Not only did they declare that their actual ability to resuscitate was low but they also declared inadequacy in their medical training programs.

**Table 2:** Sample of the responses given by the participants

Question	Correct respondents n (%)	Wrong respondents n (%)
Where do we resuscitate	174 (91)	18 (9)
Composition of the resuscitation team	92 (48)	100 (52)
When is the neonate suctioned	61 (32)	131 (68)
When do we intubate?	122 (64)	70 (36)
How is the new-born stimulated?	54 (28)	138 (72)
What is the best source of gas for resuscitation	48 (25)	144 (75)
How is oxygen delivered and at what rate?	2 (1)	190 (99)
Use of drugs such as aminophylline, adrenaline, sodium bicarbonate, hydrocortisone	2 (1)	190 (99)
What are appropriate fluids for resuscitation?	1 (1)	191 (99)
What is the best ventilation support of neonates during transport?	1 (1)	191 (99)

## Discussion

The observations of the current study reflect an overall poor performance amongst the health care personnel sampled on neonatal resuscitation. Similar observations have been made in developing countries, where neonatal resuscitation is a big challenge [10–12]. It may be a reflection of ill preparation of health care personnel during training. Ironically, medical officers in particular spend extensive time in school during their undergraduate medical training [13] but still don't seem to have sufficient knowledge on neonatal resuscitation.

Despite the fact that the average work experience was 9 years and questions were designed to assess basic level of knowledge, only 35.4% of the participants managed to score above the minimum competency level. One would expect better results in a population survey with such magnitude of experience. This indicates a serious deficiency in knowledge of basic maternal and neonatal care. Many health personnel have limited knowledge of neonatal resuscitation and are yet faced with high numbers of resuscitations largely poorly executed [14]. These findings reflect ailing health system and bolster the view that very few medical institutions currently provide optimal training on essentials of maternal and newborn care.

Nurses in particular regardless of whether they had a degree or certificate qualifications performed dismally. This worrying observation indicates either lack of formal training in neonatal care or disinterest in the subject. Paradoxically, previous reports indicate that nurses and midwives always perform better than clinicians on neonatal resuscitation and have been touted as the best ambassadors to community level hospitals [15, 16]. Regarding the fact that nursing education takes almost half the duration of time it takes to train doctors [17] may explain constrained time for training. Consequently, what this implies is that many neonates who may need resuscitation may end up suffering especially in rural communities.

Noticeable was that the sampled health care personnel basically from public health sector had minimal number of hours of training on neonatal resuscitation and without much practicum exposure. Further, there were hardly any refresher courses after they graduate from medical school which further complicated their lack of competency. As a result, they remain uninformed and become helpless especially in stations in which they are deployed to serve. Similar observations have been made in rural Bangladesh, Pakistan, Vietnam and India which may be indicative of a common challenge in developing nations [18–21].

Additionally, many young health care personnel remain unsupervised and unprepared to deal with approved guidelines for neonatal resuscitation. Skills that are better passed from the senior to junior colleagues with adequate interaction remain un-inculcated resulting in inadequate knowledge and professional insecurity. There is an overall lack of good will and effort on part of consultants in public health facilities to spend time in these facilities teaching, thus putting the young professional who are inherently impatient to learn (the generation X) at risk of ignorance. In deed previous studies have revealed that the best way to pass on medical knowledge and skills is through apprenticeship which apparently lacks in this setting [22–24].

It is conceivable that even if the lack of knowledge noted in this study in terms of lower scores, refresher courses and formal training programs can offset some of the gaps. There is also need to follow up the process of translation of knowledge and skills gained by the trainees into clinical practice to periodically evaluate competency.

## Conclusion

The disappointing performance of health personnel in this essential skill, underscores the urgent need for intensified training. Increasing the duration and quality of formal training should be considered during the pre-service medical education to ensure acceptable neonatal outcome.
